# Ethnobotanical study of medicinal plants in Asagirt District, Northeastern Ethiopia

**DOI:** 10.1186/s41182-023-00493-0

**Published:** 2023-01-09

**Authors:** Muhidin Tahir, Hiwot Asnake, Tadesse Beyene, Patrick Van Damme, Amin Mohammed

**Affiliations:** 1 Department of Biology, College of Natural and Computational Sciences, Oda Bultum University, P.O. Box 226, Chiro, Ethiopia; 2 Department of Biology, College of Natural and Computational Sciences, Bonga University, P.O. Box 334, Bonga, Ethiopia; 3 Department of Biology, College of Natural and Computational Sciences, Mekelle University, P.O. Box 231, Mekelle, Ethiopia; 4 Laboratory for Tropical and Subtropical Agriculture and Ethnobotany, Department of Plants and Crops, Faculty of Bio-Science Engineering, Ghent University, Coupure Links 653, 9000 Ghent, Belgium; 5 Faculty of Tropical AgriSciences, Czech University of Life Sciences Prague, Kamycka 129, 165 21 Prague 6-Suchdol, Czech Republic; 6 Department of Plant Science, College of Agriculture, Oda Bultum University, P.O. Box 226, Chiro, Ethiopia

**Keywords:** Asagirt District, Conservation, Ethiopia, Ethnobotany, Indigenous knowledge, Medicinal plants

## Abstract

**Background:**

The people in Ethiopia have developed their own specific knowledge to use, manage and conserve plant resources, giving traditional medicine its diverse nature. Documenting and investigating the traditional and cultural use of remedial plants is vital to extract bioactive chemicals and preserve plant species. This research was conducted with the aim of documenting ethnobotanical and associated knowledge on medicinal plants in Asagirt District, northeastern Ethiopia.

**Methodology:**

The study was conducted from September 27, 2018, to April 9, 2019. A total of 367 informants (244 males and 123 females) were involved in the interviews. General informants (*n* = 349) were randomly selected, whereas key informants (*n* = 18) were selected purposively. Data were collected by using semistructured interviews, group discussions and guided field walks. We performed direct matrix ranking and preference ranking, and calculated the fidelity level and informant consensus factor (ICF). Descriptive statistics, including analysis of variance (ANOVA) and independent sample *t*-test were used to analyse the data.

**Results:**

Overall, 103 medicinal plant species belonging to 96 genera under 45 plant families were recorded to be used by Asagirt people to alleviate different health problems. The species used to heal human diseases only were (64%, 66 species) followed by both livestock and human ailments (31%, 32 species) and livestock diseases only (5%, 5 species). Asteraceae and Fabaceae were best-represented (10.7%, 11 species each). The most frequently used plant parts were leaves (28%, 29 species), followed by seeds (16%, 17 species). The most important method of herbal remedy preparation was crushing (20.4%, 21 species). The common route of administration was oral (46.6%, 48 species), followed by dermal (22.3%, 23 species). Febrile illness, fever, headache, amoebiasis, typhoid and diarrhoea ailment categories had the highest ICF value (0.99). *Ocimum lamiifolium* Hochst. ex Benth. scored the maximum fidelity level value (98%).

**Conclusion:**

Asagirt District is comparatively rich in medicinal plants and their associated knowledge. However, firewood collection, construction, the expansion of agricultural activities and timber production are the major challenges to medicinal plants. Hence, joint management with the people in Asagirt District in overall medicinal plant conservation would save medicinal plant resources.

**Supplementary Information:**

The online version contains supplementary material available at 10.1186/s41182-023-00493-0.

## Background

People around the world started manipulating remedial plants before the advent of modern or allopathic medicine to prevent various ailments [1–3]. The World Health Organization (WHO) reported that 80% of populations in the world depend on plants used in traditional medicine to alleviate a wide range of diseases [4]. The indigenous knowledge on the use and preparation of plants used in traditional medicine is an important factor that helps to define the cultural identities of communities and provide evidence for links to their past [5]. Documenting traditional knowledge on plants used in traditional medicine is a vital step in obtaining new lead bioactive compounds for developing allopathic drugs [3]. It should be noted that plants produce diverse natural products, including bioactive metabolites, phenolics and antioxidants [6–10], which are important to address a wide range of diseases [11–14]. For instance, coumarins, which are commonly used in the treatment of prostate cancer [15], were first isolated from *Dipteryx odorata* (Aubl.) Willd. [16]. Ethanol extract of *Terminalia pallida* Brandis (Combretaceae) has significant antimicrobial efficacy against various bacterial and fungal strains [17]. Neuropharmacological activities of *Citrus limon* (L.) Osbeck extracts against Swiss albino mice showed a significant sleep onset reduction [18].

The indigenous people in Ethiopia have developed their own specific knowledge to use, manage and conserve plant resources, which gave traditional medicine its diverse nature [5, 19]. Approximately 6027 vascular plant species have been recorded in Ethiopia, among which nearly ten percent are endemic to the country [20, 21], and 887 have been reported to have been used to address various health problems [22, 23]. Eighty percent of the human population in Ethiopia depends on traditional remedial plants [23, 24]. In addition to inadequate provision of modern medicine, culture-associated traditions and the relatively low cost of the plants used in traditional medicine were the major factors for the high dependency on traditional remedial plants in the country [5, 25]. The diverse ethnic groups, long history of using traditional remedial plants and the existence of different topography provide Ethiopia with an enormous traditional remedial plant reservoir and indigenous knowledge [24]. However, most traditional knowledge is kept in secrecy because healers only pass on their knowledge to family members, mostly to the oldest son [26]. Besides, indigenous knowledge on plants in Ethiopia is subject to loss since it is usually transmitted orally [27]. Moreover, remedial plant resources and indigenous knowledge in the country are being lost due to economic infrastructure, rapid loss of natural sites and changes in lifestyle [28]. Besides, up to now, there is no documented ethnobotanical study regarding traditional medicinal plants and associated knowledge in Asagirt District. There is an ongoing national project on medicinal plant documentation at the national level by the Ethiopian Biodiversity Institute (EBI). Thus, this study is an input for medicinal plant protection and further phytochemical investigation. Hence, this research aimed to document medicinal plants associated with ethnobotanical knowledge in Asagirt District.

## Materials and methods

### Description of the study area

**Fig. 1:**
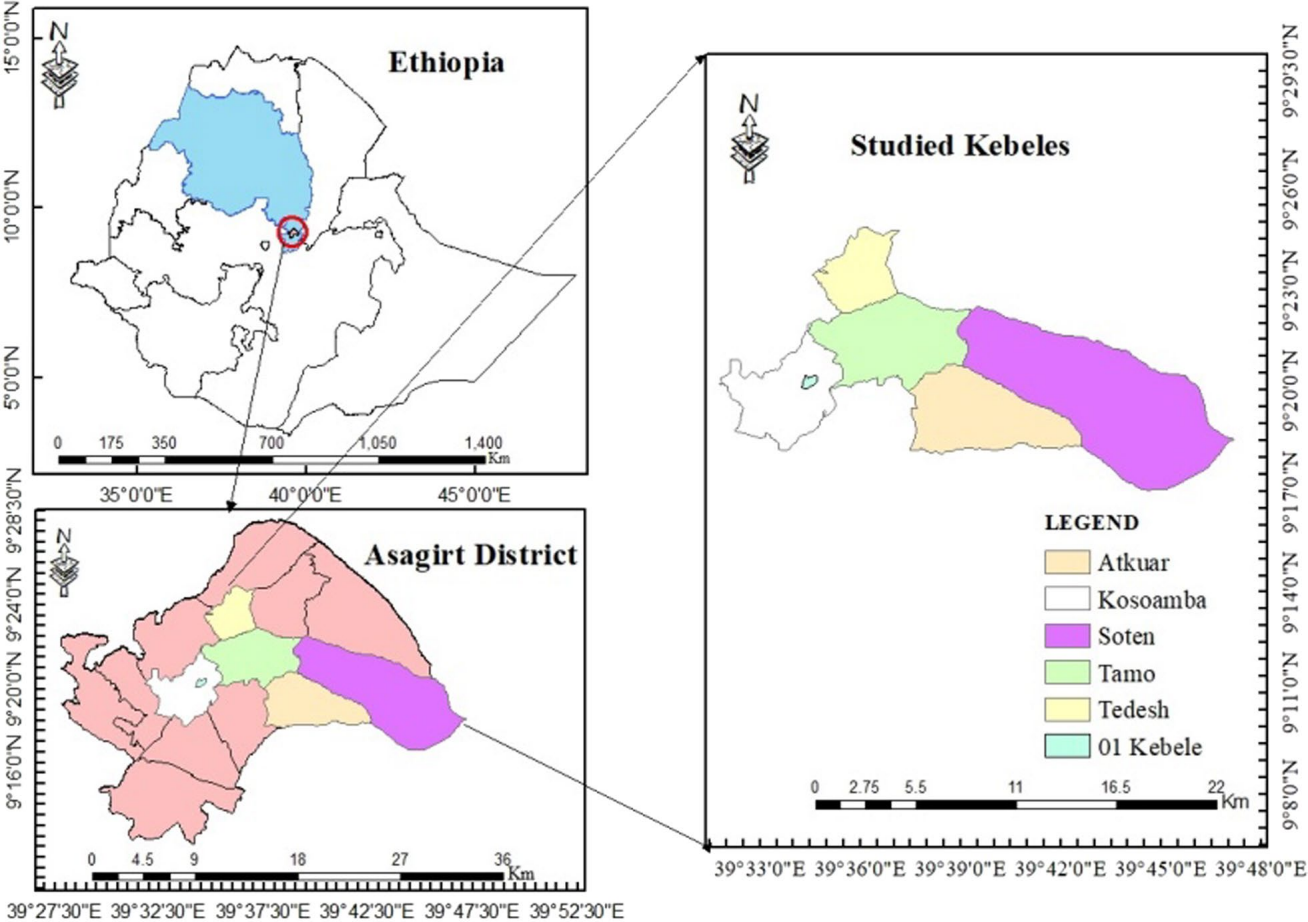
Map of Asagirt District and sampled kebeles

**Fig. 2:**
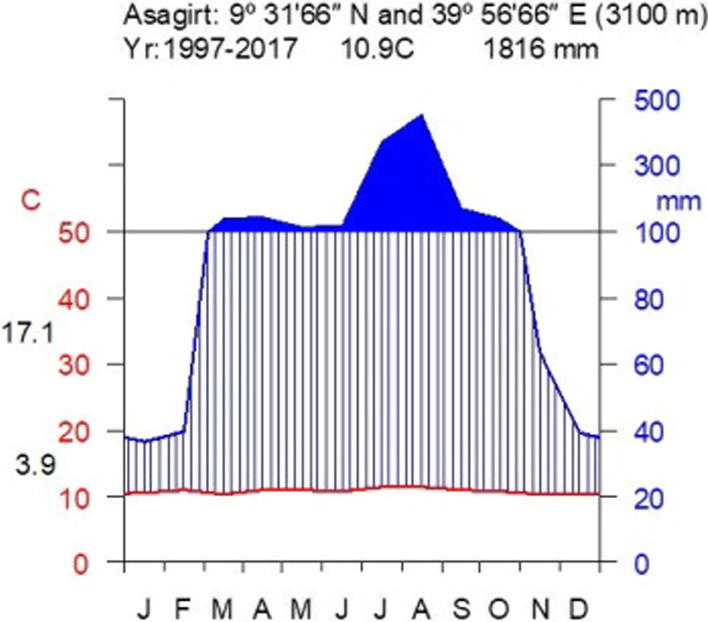
Climatic diagram showing mean annual temperature and rainfall from 1997 to 2017 (Data source: National Metrological Services Agency, 2018)

The study was conducted in Asagirt, North Shewa Zone of Amhara Region, Northeastern Ethiopia. The district is located at 9° 18′–9° 43′ N and 39° 31′–39° 62′ E, with an elevation ranging from 2231 to 3418 m a.s.l. Asagirt is bounded by Hagere Mariamna Kesem to the southwest, Angolalla Tera to the northwest, Basona Werana to the north, Ankober to the northeast, Afar Regional State to the east and Berehet District to the southeast (Fig. 1). The mean annual precipitation in Asagirt District amounts to 1816 mm. The district has unimodal rainfall with a rainy period from March to October (peak in August). The mean annual temperature of Asagirt is 10.9 °C. The maximum and minimum temperatures in the district are 17 °C and 4 °C, respectively (Fig. 2). Asagirt District has a total land area of 52,135 ha divided into 15 *kebeles* (wards/smallest administrative units). The district had a population of 48,371, of whom 24,674 were men, 23,697 were women, 1278 (2.64%) were urban inhabitants, and 47,093 (97.36%) were rural inhabitants.

### Research design

#### Sample size and informant selection

A total of six rural *kebeles,* i.e., Kosoamba, Soten, Tedesh, Tamo, Atkuar and 01 *kebele* were selected from a total of 15 *kebeles* in Asagirt District based on recommendations of local elders, local authorities, presence of traditional healers and agro-climatic conditions. Kosoamba and 01 *kebele* were selected from highland agro-climatic zones, Tedesh and Tamo from midland, and Soten and Atkuar from lowland.

To determine a representative sample for selected *kebeles* (smallest administrative unit)*,* we used Cochran’s (1977) formula as indicated by Bartlett et al. [29] as follows:

1 $$n = N/1 + N\left( e \right)^{2} ,$$

where *n* is the research sample size, *N* is the total number of households in all six selected *kebeles*, *e* is the maximum variability or margin of error 5% (0.05), and 1 is the probability of the event occurring. As a result, a total of 367 informants (244 males and 123 females) were selected using Cochran’s sample size formula.

Eighteen herbal practitioners (14 males and 4 females), two to four from each *kebele,* were selected purposefully based on their knowledge as recommended by local people. The general informants (respondents) were randomly selected.

The sample size for each *kebele* was calculated using the proportion of the number of households (HH) in the respective *kebeles*. Therefore, the total household number of Kosoamba was 987, yielding a number of 83 (*n* = 987 × 367/4373 = 83). The same calculation was applied for the other *kebeles*, resulting in 01 *kebele* (HH = 484, *n* = 41), Tedesh (HH = 595, *n* = 50), Tamo (HH = 1076, *n* = 90), Soten (HH = 565, *n* = 47) and Atkuar (HH = 666, *n* = 56). From each household, one general informant, whose age is greater than 18, was selected randomly. Four focus group discussions with a member of 8–15 key informants (traditional healers) and elderly people were conducted on selected issues such as threats, conservation status and dosage of medicinal plants following Martin [30]. Group discussions with traditional healers and the elderly people help the researcher to confirm the data collected from individuals. Information obtained during the discussions was carefully recorded and interpreted as indicated by Martin [30].

#### Data collection

Qualitative ethnobotanical data were collected from September 27, 2018, to April 9, 2019. We employed semistructured interviews to record data on medicinal plants involving the plant part used, method of preparation, route of administration, conservation and challenges to medicinal plants following Martin [30]. Field walks were conducted to note the habits, habitats and appearance of medicinal plants. In addition, voucher specimens were collected, and recordings that explain the plant species were made at their location.

We identified plant species in the field, herbarium, with expert assistance, using taxonomic literature, comparison with authentic specimens and various volumes of books on the flora of Ethiopia and Eritrea (FEE) [31–38].

### Data analysis

#### Direct matrix ranking

Direct matrix ranking of six multiuse plants used in traditional medicine by four key informants was conducted in Asagirt District, as shown by Martin [30], to compare multipurpose medicinal plant species and the degree of their use. Depending on the data collected from respondents, multiuse species in Asagirt District were chosen. The use diversities of these plants for different purposes, such as charcoal, furniture, medicine, firewood, fencing, and construction material were presented to four key respondents to give values; consequently, the values given by each key respondent were summed up and ranked. To evaluate the reliability of information recorded during the interview, informants were contacted at least two times for the same ideas, and the validity of the information was proven and recorded following the method adopted by Alexiades [39].

#### Preference ranking

If different species are given to a similar health problem, people show preference for one over the other. Hence, preference ranking was performed for the most vital medicinal plants used to treat specific diseases based on Martin’s [30] method. The selected key informants assigned values from 0 to 7 for the most preferred to the least preferred plant species against certain diseases. The values were then summed, and ranks were assigned to each plant species.

#### Informants consensus factor

We calculated the informant consensus factor (ICF) for each group of diseases to identify the agreement of the respondents on the reported remedy for the disease categories [40]. Diseases in Asagirt District were classified based on the International Classification of Primary Care (ICPC-2, 2003), as indicated by Staub et al. [41] based on affected organs as a criterion. It was calculated using the formula:

2 $${\text{ICF}} = {\text{Nur}}{-}{\text{Nt}}/{\text{Nur}}{-}1,$$

where Nur is the number of use reports for a particular ailment category and Nt is the number of medicinal plant species used for a particular ailment category by all informants.

#### Fidelity level

The relative curing potential of each plant species used to heal human disease was computed [40]. Its formula is given as follows:

3 $${\text{FL}}\% = I_{{\text{p}}} /I_{{\text{u}}} \times 100,$$

where FL% is the percentage of the fidelity level, *I*_p_ is the number of respondents who indicated the use of a species for the same major diseases and *I*_u_ is the total number of respondents who mentioned the plant for any major ailments indicated. The total use and specific use reports of medicinal plant species by respondents for disease treatment were recorded, and the fidelity level was calculated and summarized.

Medicinal plant knowledge comparison between different social groups in Asagirt District was performed using SPSS (version 20). ANOVA (one-way analysis of variance) was performed to check the mean knowledge significant difference on medicinal plants mentioned by different social groups. To determine medicinal plant knowledge differences between marital status (married and single) and healing experience (key informants and general informants), a *t*-test was carried out. Medicinal plant knowledge was determined in terms of the number of medicinal plants mentioned by different respondents. Microsoft (2010) Spreadsheet Excel was used to calculate sums and percentages and to tabulate and draw graphs.

## Results

### Sociodemographic characteristics of respondents

Out of a total of 367 informants involved in this study, 67% (*n* = 244) were men, whereas 33% (*n* = 123) were women. Regarding marital status, the majority of the informants were married (83%, 306), followed by single (17%, 61). The age of the informants included in the study ranged from 18 to 80. Most of the informants were aged 61–80 (43%, 159), followed by 41–60 (33%, 120). The education levels of the informants ranged from illiteracy to college level. Most of the informants were illiterate (60%, 221), followed by elementary school (30%, 108) (Table 1).

**Table 1:** Medicinal plant knowledge among different social groups of the study area (*n* = 367)

Parameter	Category	*N*	Mean	*p* value
Healing experience	Key informants	18	6	0.0001
	General informants	349	3.3	
Age groups (in years)	18–40	88	4.9	0.001*
	41–60	120	9.8	
	> 61	159	16.0	
Education level	College	9	3.6	0.001
	High school	28	4.8	
	Elementary school	108	10.7	
	Illiterate	221	12.8	

*Shows a significant difference at (*p* < 0.05) between averages of the paired categories

A significant difference (*p* < 0.05) between herbal practitioners and general respondents was observed in the mean number of medicinal plants known and used in the Asagirt District: herbal practitioners were more knowledgeable (27.8) than general respondents (9.4). Likewise, comparison of knowledge between married and singles showed that there was a significant mean knowledge difference of medicinal plants reported between the two groups (*p* < 0.05). One-way analysis of variance (ANOVA) showed that there was a significant difference (*p* < 0.05) in the mean number of medicinal plant species reported between different age groups. There was also a significant difference (*p* < 0.05) in the average number of medicinal plants reported among different education levels (Table 1).

### Medicinal plant diversity and their growth forms

A total of 103 medicinal plant species belonging to 96 genera in 45 plant families were documented to be used by the people of Asagirt District. The species used to heal human diseases only were (64%, 66 species) followed by both livestock and human ailments (31%, 32 species) and livestock diseases only (5%, 5 species) (see Additional file 1). Asteraceae and Fabaceae were the best-represented families (10.7%, 11 species each), followed by the families Solanaceae (8.7%, 9 species), Lamiaceae (6.8%, 7 species) and Euphorbiaceae (5%, 5 species) (Table 2). Herbs constituted the largest number of growth forms (44%, 45 species), followed by shrubs (38%, 39 species), trees (15%, 16) and climbers (3%, 3) (see Additional file 1).

**Table 2:** Number of medicinal plants and genera belonging to each family

Family	Number of species	% of species	Number of genera
Asteraceae	11	10.7	9
Fabaceae	11	10.7	11
Solanaceae	9	8.7	8
Lamiaceae	7	6.8	7
Euphorbiaceae	5	4.9	5
Cucurbitaceae	4	3.9	4
Boraginaceae	3	2.9	3
Brassicaceae	3	2.9	3
Myrtaceae	3	2.9	3
Rosaceae	3	2.9	3
Rutaceae	3	2.9	2
Other	41	39.8	38

### Plant parts used, modes of preparation and administration

**Fig. 3:**
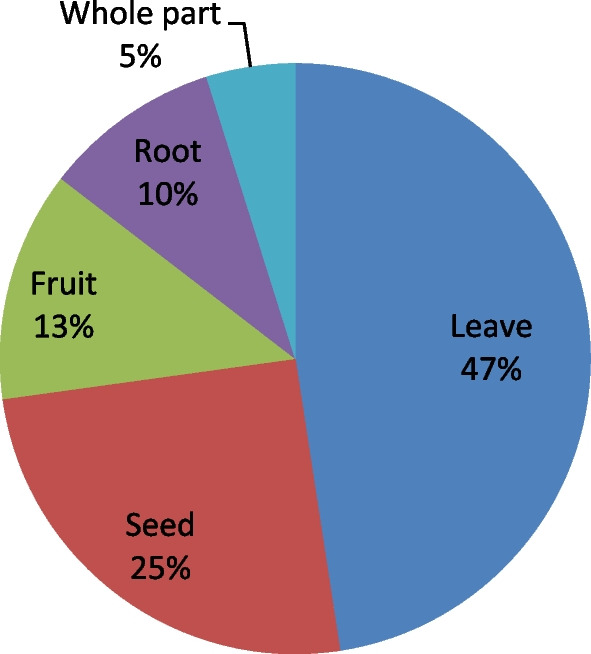
Parts of medicinal plants used by the local people in the study district

**Fig. 4:**
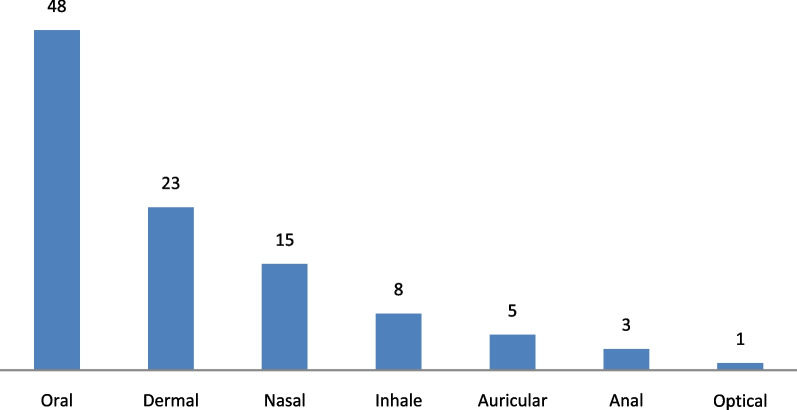
Route of administration

Out of a total of 103 medicinal plant species, leaves were the most widely used plant parts (47%, 49 species), followed by seeds (25%, 26 species) (Fig. 3). The local people in Asagirt District use different methods and conditions of preparation. The highest method of preparation was crushing (20.3%, 21 species), followed by squeezing (12.6%, 13 species) (see Additional file 2). Fresh form was the dominant condition of preparation (50%, 52 species) followed by both dried and fresh form (33%, 34 species) and dried (17%, 17 species). Different ways of administration of the prepared remedies to treat various ailments were observed. Oral administration was the principal route of administration (46.6%, 48 species), followed by skin application (22.3%, 23 species) (Fig. 4). Focus group discussions made with the traditional healers and elderly people in Asagirt District showed that there was no treatment that was taken internally by pregnant women and children less than 6 months.

### Human and livestock ailments treated

A total of 81 human diseases were treated by 66 plant species. The most common human diseases were tonsillitis, wounds, constipation, dandruff, common cold, haemorrhoid, ascariasis, cough, gastritis, abdominal pain, diarrhoea, febrile illness and hypertension (see Additional file 1). Herbal practitioners were also visited frequently for different diseases, including febrile illness or “*Mich*” and spider poison. The local community prefers traditional healers for such diseases rather than modern medication. The numbers of ethnoveterinary important plant species that were used to treat livestock ailments recorded in Asagirt District were five, composed of five genera under four families. Four species were obtained from the home gardens, while one species was found in the wild. Diseases such as anthrax, leech, blackleg, cough, diarrhoea, helminthiasis, wounds, abdominal plotting, lice infestation in chickens, lumpy skin disease and poor appetite are common livestock ailments in Asagirt District. A number of livestock ailments can be cured by a single plant, whereas a number of plants can heal a single ailment. For instance, anthrax and leech were reported to be treated by five medicinal plant species each (see Additional file 1).

Medicinal plants that were assumed to be effective and consistent in treating diseases in groups of ailments obtained higher values of informant consensus factor. Plants used against two categories of health problems, febrile illness, fever and headache, amoebiasis, typhoid and diarrhoea had the highest ICF value (0.99) each, followed by medicinal plants used against body swelling, abscess and cellulites (0.98) (Table 3).

**Table 3:** Informant consensus factors for 12 categories of ailments

Ailment category	Plant species	Use citation	ICF
Febrile illness, fever and headache	8	816	0.99
Amoebiasis, typhoid and diarrhoea	6	543	0.99
Body swelling, abscess and cellulites	4	262	0.98
Epistaxis and bleeding	5	193	0.98
Eye problem, ear problem and expel ear mite	5	166	0.97
Asthma, common cold and cough	11	360	0.97
Fire burn, wound, tonsillitis and toothache	12	480	0.97
Ascariasis, taeniasis and tape worm	11	319	0.96
Gastritis, stomachache, constipation and vomiting	19	532	0.96
Rabies and snake bite	5	92	0.95
Ringworm, scabies, dandruff, lumpy skin disease, mumps, skin disease, itching, tinea versicolor and impetigo	16	361	0.95
Liver problem, hepatitis and diuretic	5	62	0.93

A fidelity level of six remedial plants showed that *O. lamiifolium* was the most frequently utilized for the treatment of febrile illness, accounting for 98%, followed by *S. incanum* (93%), for tonsillitis (Table 4).

**Table 4:** Fidelity level of six medicinal plant species

Scientific name	Main ailment treated	*I*_p_	*I*_u_	FL	FL%
*Coffea arabica* L.	Wound	15	16	0.94	94
*Kalanchoe marmorata* Baker	Common cold	132	200	0.66	66
*Ocimum lamiifolium* Hochst. ex Benth.	Febrile illness	163	165	0.99	99
*Olea europaea* L.	Toothache	13	14	0.93	93
*Solanum incanum* L.	Tonsillitis	135	139	0.97	97
*Solanum nigrum* L.	Eczema	4	5	0.8	80

The herbal practitioners of Asagirt District showed a preference for medicinal plants for a particular ailment. The preference ranking of eight medicinal plants that were reported for curing tonsillitis by ten key informants showed that *S. incanum* stood first and hence is the most effective medicinal plant to cure tonsillitis, accounting for 62, followed by *R. nepalensis* (57) (Table 5).

**Table 5:** Preference ranking of eight medicinal plants in treating tonsillitis

Key informants	Medicinal plants							
	*Schinus molle* L.	*Gossypium barbadense* L.	*Kalanchoe petitiana* A.Rich	*Leonotis ocymifolia* (Burm.f.) Iwarsson	*Olea europaea* L.	*Rhamnus prinoides* L’Herit	*Rumex nepalensis* Spreng	*Solanum incanum* L.
K_1_	5	4	2	1	6	3	8	7
K_2_	3	1	8	4	5	2	7	6
K_3_	1	3	4	8	2	7	6	5
K_4_	2	3	7	1	8	6	5	4
K_5_	8	1	7	3	2	5	4	6
K_6_	5	4	6	2	3	1	7	8
K_7_	7	6	4	3	2	1	5	8
K_8_	6	2	5	1	8	3	7	4
K_9_	4	7	5	2	3	1	6	8
K_10_	1	5	3	4	7	8	2	6
Total	42	36	51	29	46	37	57	62
Rank	5th	7th	3rd	8th	4th	6th	2nd	1st

Six commonly cited multiuse species for seven use groups based on the information collected from four key informants were included in the direct matrix ranking. The species *Cordia africana* Lam. ranked first and hence is the best preferred plant by local people for various uses, accounting for 120, followed by the species *Croton macrostachyus* Hochst. ex Delile 91 (see Additional file 3).

The results of this study showed that some medicinal plants were more popular than others. In this study, the highest informant consensus was found for *O. lamiifolium*, cited by 316 (8%) informants, followed by *S. simensis* 303 (7.6%) (Table 6).

**Table 6:** List of medicinal plants cited by the highest number of informants

Scientific name	Number	Percent (%)
*Ocimum lamiifolium* Hochst. ex Benth.	316	7.99
*Satureja simensis* (Benth.) Briq.	303	7.66
*Ruta chalepensis* L.	231	5.84
*Kalanchoe petitiana* A.Rich	223	5.64
*Allium sativum* L.	222	5.61
*Withania somnifera* (L.) Dunal	222	5.61
*Urtica simensis* Hochst. ex A.Rich.	222	5.61
*Zingiber officinale* Roscoe	222	5.61
*Echinops kebericho* Mesfin	222	5.61
*Guizotia abyssinica* (L.f.) Cass.	123	3.11
*Lepidium sativum* L.	120	3.03
*Solanum incanum* L.	103	2.60

In Asagirt, indigenous knowledge on remedial plants was obtained through various acquisition methods. The highest medicinal plant knowledge acquisition from the local communities was from parents (72.8%), followed by observation (19.6%), elders (7.6%) and others (6.5%).

### Threats to medicinal plants and use

Various factors were recorded as the main conservation challenges to medicinal plant species in Asagirt District. The highly cited threat by respondents was agricultural expansion, which accounted for 65%, followed by firewood (15%), charcoal production (10%), timber production and construction (5%).

Focus group discussions made in the study area showed that deforestation for agriculture was the most threatening factor for medicinal plants, particularly multipurpose species that are highly affected. For example, *C. africana* was used for different purposes, including construction, timber production, fuel and medicinal purposes, by local people of Asagirt District that will result in the loss of this plant if it continues unabated.

Home gardens are a good place for protecting medicinal plant species and for better transfer of indigenous knowledge to younger generations. Some traditional practitioners were involved in medicinal plant conservation by cultivation in the village. Most medicinal plants in Asagirt (81%) were reported to be cultivated in home gardens (see Additional file 1). The people in Asagirt manage plant resources for their medicinal attributes. Some healers of Asagirt District grow medicinal plants with coffee plantations in home gardens and as a fence in residential places. This paves the way to pass knowledge on medicinal plants for the next generation.

Furthermore, the local people recommended some methods to conserve and protect medicinal plant species. Accordingly, the highest conservation method recommended by the local people was home gardens, mentioned by 53.4% (*n* = 196) informants, followed by plantation in mosques and churches (23%, 84), reforestation and awareness raising (10%, 37), afforestation (7%, 27) and protected areas (6%, 23).

## Discussion

### Sociodemographic characteristics of respondents

Herbal practitioners in Asagirt District were more knowledgeable than general respondents (informants) (Table 1). This could be related to their years’ long experience and high level of secrecy in using remedial plants [24]. A similar finding was also reported by Giday et al. [42] in that herbal practitioners were more knowledgeable in the number of reported medicinal plants than general informants. However, the finding by Demie et al. [43] showed insignificant knowledge differences between key and general informants on medicinal plants used.

Comparison of knowledge between married and single participants showed that married respondents tended to mention a higher number of medicinal plants. The study performed by Kidane et al. [25] showed that married respondents were more knowledgeable than single respondents. The significant differences (*p* < 0.05) in knowledge of remedial plants among age classes indicated that the knowledge of remedial plants among younger people was low. Older people could have a high opportunity for more cultural exchange and practice with remedial plants than younger people [24]. Similar findings were reported in other parts of the country [3, 26, 42, 43]. In Asagirt District, lower grade people and illiterate people were more knowledgeable (*p* < 0.05) than educated people. The impact of modern education on the knowledge of traditional medicine might be the reason for the lower knowledge of educated people [26]. This result agrees with the studies performed in Ethiopia [3, 25, 28], which indicated that illiterate informants had better knowledge of medicinal plants.

### Medicinal plant diversity and their growth forms

In Asagirt District, the local people highly depend on diverse plant species for medicinal value (*n* = 103). The number of medicinal plant species documented in Asagirt District was higher than that from similar ethnobotanical studies in Ethiopia, i.e., in Babile District, 51 species were mentioned [44], in Ghimbi District *n* = 49 [45], in Dirre Sheikh Hussein heritage site *n* = 87 [43], Suro Barguda District *n* = 98 [46] and Sheko ethnic group *n* = 71 [28]. In addition to inadequate provision of modern medicine, culture-associated tradition and the relatively low cost of the plants used in traditional medicine might be the major factors for the high utilization of the plants used in traditional medicine, as also confirmed in other parts of Ethiopia [24].

The occurrence of a higher number of plants from the families Asteraceae and Fabaceae might be related to the wider distribution of the species under these families in a wide range of elevations [47]. This finding is in line with results reported in Ethiopia [3, 25, 45, 48, 49] as well as other countries of the world [50, 51].

The recorded higher number of plant species for human remedy in Asagirt District might be due to the prevalence of human diseases such as tonsillitis, wound, constipation, dandruff, common cold, haemorrhoid, ascariasis, cough, gastritis, abdominal pain, diarrhoea, febrile illness and hypertension, intestinal parasites and amoebiasis (see Additional file 1).

The high use of herbs in Asagirt might be because of the easy availability near the village to Asagirt people, as also shown by Lulekal et al. [52] in their study area. In the same way, most studies in Ethiopia [3, 25, 53] and elsewhere in the world [54–56] confirmed the dominance of herbs. However, the results reported from the Babile District [44], eastern Ethiopia, and the Afar Region, Ada’a District [53], indicated that shrubs were the most widely used habit (growth form) of medicinal plants.

### Part used, method of preparation and administration

The wide use of leaves in Asagirt District might be due to the effectiveness of bioactive ingredients in their parts and better accessibility, as also indicated by Tahir et al. [24] in their study area. Harvesting the leaves has less influence on the survival of the plant species, whereas using roots affects the survival of the plants [56–58]. This result agrees with most studies in Ethiopia [43, 59, 60] and in the world [61–63]. Nevertheless, the result reported from Ada’a, Afar region of western Ethiopia [53], showed that roots were found to be the most frequently used plant parts.

The higher amount of crushing in medicinal plant preparation in Asagirt might be due to simple preparation at any place using stones most times, which could be done by most local community members, as also indicated by Tahir et al. [24]. Informants stated that the crushed remedial plant parts that are drenched in water lead to an effective and fast response to health problems. Similarly, the findings by Belayneh et al. [44] and Megersa et al. [64] indicated crushing as a better method of remedy preparation.

Some plants do have different application and preparation methods for different disease types of human ailments in the Asagirt District. For example, the leaves of *S. simensis* were crushed, squeezed and smoked. Drinking the squeezed leaves of this species was used to cure headache, sniffing the smoke was used to cure sudden sickness, and creaming on the body was used to cure high fever for human ailments (see Additional file 1).

The wide harvesting of fresh plant parts could be due to the high efficacy of their bioactive components, which could be lost upon drying [53]. Likewise, ethnobotanical studies conducted in the country also showed a preference for fresh plant parts [26, 45, 65, 66]. However, the recurrent use of fresh materials could soon minimize the chance of preservation for later use [45].

Oral administration was the dominant mode of administration, indicating a higher prevalence of internal ailments in Asagirt District. However, the dose should be given greater care in the oral system than in the dermal system since it might cause severe internal problems. Similarly, various research findings in Ethiopia [3, 53] as well as other countries in the world [1, 55] mentioned oral application as the primary method of administration. However, Giday et al. [42] in their studies in southwest Ethiopia reported that most of the Bench herbal remedies were applied on skin.

The highest recorded ICF value (0.9) in the study area indicates the best agreement among the informants on the use of medicinal plant species for treating febrile illness, fever, headache, amoebiasis, typhoid and diarrhoea. The highest informants’ agreement together with high use reports for these groups of ailments could also show a high prevalence of the categorized ailments [24]. In addition, higher ICF values for diseases such as febrile illness, fever and headache and amoebiasis, typhoid and diarrhoea might be indicative of the presence of similar ethnomedicinal plant knowledge [47, 64, 67] and their continued usage in similar ways among community members [68]. A high informant consensus factor (ICF) value is vital to distinguish plants that have probably higher bioactive components [40]. The highest ICF value for febrile illness was reported from Ganta Afeshum, northern Ethiopia [25].

Direct matrix ranking analysis indicated that *C. africana*, *C. macrostachyus* and *Afrocarpus falcatus* (Thunb.) C.N.Page were the most preferred multipurpose plant species in Asagirt District. Nonetheless, these plants were exploited for their use other than medicinal purposes as well as for medicinal value. Hence, such plant species, particularly top-ranked species such as *C. africana*, are supposed to be the most threatened species in the near future. Hence, this finding calls for joint conservation action to protect multiuse plant species. Ethnobotanical investigations performed in Ethiopia [24, 25, 47, 64] also indicated that *C. africana* and *A. falcatus* were multipurpose medicinal plants in their study area.

The highest fidelity level values for *O. lamiifolium* (98%) against febrile and *S. incanum* (93%) against tonsillitis could be taken as a clue for the high curative potential of these plants against the corresponding diseases. Plants with the highest fidelity level values can be taken as a clue for phytoextraction studies to prove the efficacy of bioactive ingredients [40, 67]. A similar result was reported by Staub et al. [41] in that *O. lamiifolium* has the highest fidelity level against febrile disease.

Antimicrobial activities and chemical compositions of *O. lamiifolium* [69, 70], *R. chalepensis*[71], *K. petitiana*, *L. sativum* [72] and *S. incanum* [72] were indicated elsewhere in the world. In Asagirt District, some plants that have been tested for their therapeutic action were *Glycine max* (L.) Merr., *Gossypium* spp., and *Capsicum frutescens* Rodsch against diabetes [73–75], *Trigonella foenum-graecum* L. for inducing labour and adding digestion [76] and *Citrus aurantiifolia* (Christm.) Swingle against obesity [77].

The highest knowledge on traditional remedial plants in Asagirt district was acquired from parents, indicating that most of the knowledge acquisition from the local community was in a secret manner. In addition, the indigenous knowledge of rural people in Asagirt District was orally transferred to the family member. Hence, the transferring system could be subjected to loss due to secrecy and oral transfer of the indigenous knowledge within the family member [24]. Similar findings were reported from Ethiopia and other countries [23, 25, 63, 78].

### Threats to medicinal plant knowledge and use

Agricultural expansion was the highest threat to plants used in traditional medicine mentioned by respondents in Asagirt District, followed by the collection of plants for firewood. Focus group discussion showed that deforestation for agriculture was the highest mentioned conservation challenge to medicinal plants in Asagirt. Similar results in Ethiopia were reported [3, 24, 64] in that agricultural expansion and collection of timber wood were the main conservation challenges of medicinal plant resources. Agricultural expansion is the main driver for the loss of plants used in traditional medicine in Ethiopia [45, 47] because most of the communities in the country highly depend on agriculture as their main economic activity with limited landholding and high human population. Informants reported that the Youngs refused to know or use traditional medicine; hence, much invaluable information could be lost whenever traditional medicinal practitioners die without sharing their knowledge with others.

Most of the informants in Asagirt District recommended conserving medicinal plants in the home garden, planting them in mosques and churches, and raising awareness are the best conservation approaches for the plants used in traditional medicine. In Asagirt, community-based governmental biodiversity conservation practices were employed to maintain the sustainable use of their products. Cultivating plants used in traditional medicine in natural wild settings and home gardens is vital to improve future access for the healthcare of rural people and further laboratory investigation [24].

## Conclusions

The people in Asagirt are relatively rich in traditional knowledge on remedial plants, which is still maintained among local people. Overall, 103 medicinal plant species were noted to treat humans, livestock, and both human and livestock ailments. There are locally preferable treatments by traditional healers for some diseases in the area, such as febrile illness, sudden sickness and spider poison, compared to modern clinics. Greater preference for species such as *S. incanum*, *R. nepalensis*, *K. petitiana* and *O. europaea* in treating tonsillitis might be an indication for further phytochemical analysis, pharmacological investigation and conservation measures.

Threats that come to medicinal plant resources due to overutilization, such as agricultural expansion, decrease medicinal plant species, whereas threats resulting from secrecy, oral-based knowledge transfer, and the reluctance of the young generation to gain knowledge erode knowledge on medicinal plants. Therefore, awareness creation should be facilitated to conserve and preserve medicinal plants and indigenous knowledge, particularly the species that are harvested frequently for their roots, such as *Crinum abyssinicum* Hochst. ex A.Rich., and *Rumex nervosus* Vahl. The communities in Asagirt should participate in the preservation, management and conservation of plant resources used in medicine and the associated knowledge in their locality. Cultivating plants used for multiple purposes, such as *C. africana*, should be facilitated. The development of community-based forest priority areas in the district for conserving forests, particularly medicinal plant resources in Asagirt, should be encouraged.

## Supplementary Information

**Additional file 1.** List of medicinal plants, habits, part used, condition and ways of preparation, routes of administration, ailment treated and collection number in Asagirt District (CP = Condition of preparation, MA = Method of application, RA = Routes of administration, H = Home garden, W = Wild, F = Fresh, D = Dried, H/L = Human or Livestock, HU = Human, Li = Livestock Fu = Furniture, Fr = Forage, Fo = Food, Sp = Spice, Co = Construction, Or = Ornamental, Fe = Fuel, Si = Soil fertility, So = Social use, Tb = Traditional beverage, Ch = Charcoal, L = Live fence).

**Additional file 2.** Methods used in the preparation of remedies.

**Additional file 3.** Direct matrix ranking of six medicinal plant species by four informants based on seven use criteria.

## Data Availability

All the data collected for this study were analysed, interpreted, and included in this manuscript, and its supplementary materials are attached as Additional files 1, 2 and 3.

## Abbreviations

HH: Household
ICF: Informant consensus factor
WHO: World Health Organization
