# Supplementary material for: Ethnobotanical study of medicinal plants in Asagirt District, Northeastern Ethiopia
Source: Trop Med Health. 2023 Jan 9;51:1. doi: 10.1186/s41182-023-00493-0 (PMC9827656; doi:10.1186/s41182-023-00493-0)
Supplement: Supplementary file 1 — Additional file 1. List of medicinal plants, habits, part used, condition and ways of preparation, routes of administration, ailment treated and collection number in Asagirt District (CP = Condition of preparation, MA = Method of application, RA = Routes of administration, H = Home garden, W = Wild, F = Fresh, D = Dried, H/L = Human or Livestock, HU = Human, Li = Livestock Fu = Furniture, Fr = Forage, Fo = Food, Sp = Spice, Co = Construction, Or = Ornamental, Fe = Fuel, Si = Soil fertility, So = Social use, Tb = Traditional beverage, Ch = Charcoal, L = Live fence). [file 41182_2023_493_MOESM1_ESM.docx]

**Additional file 1** List of medicinal plants, habits, part used, condition and ways of preparation, routes of administration, ailment treated and collection number in Asagirt District (CP = Condition of preparation, MA = Method of application, RA = Routes of administration, H = Home garden, W= Wild, F= Fresh, D= Dried, H/L= Human or Livestock, HU = Human, Li = Livestock Fu = Furniture, Fr = Forage, Fo= Food, Sp = Spice, Co = Construction, Or = Ornamental, Fe = Fuel, Si= Soil fertility, So = Social use, Tb = Traditional beverage, Ch = Charcoal, L = Live fence).

| Scientific name | Family | Local name | Source | Habits | Part used | CP | Used by | Disease treated | Methods of preparation | MA | RA | Other use | Collection number |
| --- | --- | --- | --- | --- | --- | --- | --- | --- | --- | --- | --- | --- | --- |
| *Acacia abyssinica* Benth. | Fabaceae | Girar | H | Tree | Seed | D | HU | Sexually transmitted diseases | Seed is crushed, powdered, mixed with little water and drunk with tea cup | Drinking | Oral | Fu, Co, Fr,L, Ch, | HA 011 |
| Achyranthes aspera L. | Amaranthaceae | Tilenj | H | Herb | Root | F | HU | Stomachache | Root is chewed and swallowed during feeling of ache with coffee cup | Swallowing | Oral |  | HA 008 |
|  |  |  |  |  | Leaf | F | HU | Wound | Leaf is ground and then creamed on the affected part with finger line and tied on hand by clean cloth | Creaming | Dermal |  |  |
|  |  |  |  |  | Leaf | F | HU | Eye problem | Leaf is ground and immersed to water and the squeezed is dropped in to eye with cotton | Dropping | Optical |  |  |
| *Afrocarpus falcatus* (Thunb.) C.N.Page | Podocapaceae | Zigba | W | Tree | Leaf | F | HU | Sudden sickness | Leaf is squeezed and drunk with coffee cup | Drinking | Oral | Fu, Fe, Ch, L, Co | HA 088 |
| *Allium cepa* L. | Alliaceae | Key shenkurt | H | Herb | Seed | F | Hu | Hypertension | Seed is crushed and immersed in little water for one day and then filtrated by sieve and drunk concoction before food with teacup | Drinking | Oral | Fo | HA 012 |
|  |  |  |  |  | Seed | F | Hu | Hepatitis | Seed is chopped, eaten with *Injera* and then after honey is taken before taking other food | Eating | Oral |  |  |
|  |  |  |  |  | Whole parts | F | Li | Bloating | Whole parts are pounded, mixed with little water and then only the pure solution is drunk with coffee cup | Drinking | Oral |  |  |
| *Allium sativum* L. | Alliaceae | Nech shenkurt | H | Herb | Bulb | D | Hu | Malaria | Bulb of *A. sativum* with *L. sativum* is pounded, powdered and mixed with little water or eaten by *injera* | Eating | Oral | Fo | HA 025 |
|  |  |  |  |  | Bulb | F | Hu | Cough | Bulb is boiled with tea and drunk with tea cup before cooled | Drinking | Oral |  |  |
|  |  |  |  |  | Bulb | F | Hu | Common cold | Bulb is boiled with tea and drunk with tea cup before cooled with tea cup | Drinking | Oral |  |  |
|  |  |  |  |  |  | F | Li | Lice infestation in chicken | Juice is extracted and then creamed on the infected part | Creaming | Dermal |  |  |
|  |  |  |  |  | Leaf | F | Li | Helminthiasis | Leaf is pounded, mixed with cold water and then given to the animal to drink it | Drinking | Oral |  |  |
|  |  |  |  |  | Bulb | F | Hu | Abdominal pain | The bulb of *A. sativum* is mixed with *C. annum* and eaten | Drinking | Oral |  |  |
|  |  |  |  |  | Bulb | F | Hu | Alopecia | Bulb is rubbed on the lost part of the hair | Creaming | Dermal |  |  |
|  |  |  |  |  | Leaf | F | Li | Cough | Leaf of *A. sativum*, *S. macrosolen* and the seed of *L. sativum* are ground and mixed with water and then given for donkey with can | Drinking | Oral |  |  |
| *Aloe percrassa* Tod. | Aloaceae | Ert | H | Herb | Sap | F | HU | Hemorrhoids | Leaf latex is extracted and applied on the infected part | Creaming | Dermal |  | HA 002 |
|  |  |  |  |  | Sap | F | HU | Foreign body "(*Gudeff)*" | Leaf latex is extracted and applied on the injured part | Creaming | Dermal |  |  |
|  |  |  |  |  | Sap | F | HU | Dandruff | Leaf latex is extracted and applied on the infected part | Creaming | Dermal |  |  |
| *Anthemis tigrensis* J.Gay ex A.Rich. | Asteraceae | Chemekot | H | Herb | Leaf | F | HU | To stop bleeding | Leaf is crushed with a finger and put on the bleeding site | Put on | Dermal |  | HA 055 |
| *Artemisia abyssinica* Sch.Bip. ex A.Rich. | Asteraceae | Arit | H | Herb | Leaf | D | HU | Stomach ache | Leaf is crushed and then mixed with water then drank it with coffee cup | Drinking | Oral |  | HA 039 |
| *Artemisia afra* Jack. Ex Willd. | Asteraceae | Chigugn | H | Herb | All part | F/D | HU | Evil eye | All part are crushed, powdered and then sniffed, drunk with coffee cup and fumigated | Inhale, Drinking and creaming | Oral, nasal & dermal |  | HA 061 |
|  |  |  |  |  | Leaf | F | HU | Tinea versicolor | Leaf is crushed, squeezed and mixed with the juice of lemon then creamed the infected part | Creaming | Dermal |  |  |
|  |  |  |  |  | Leaf | D | HU | "*Dorokenken*" | Leaf is put on the fire and smoked in the house | Inhale | Nasal |  |  |
| *Asparagus africanus* Lam. | Asparagaceae | Seriti | W | Shrub | Whole part | F | HU | Eczema "(*Chiffi*)" | Leaf is crushed, squeezed and mixed with crushed, squeezed leaf of *T. indica* and then creamed the infected part | Creaming | Dermal |  | HA 069 |
| *Astragalus atropilosulus* (Hochst.) Bunge | Fabaceae | Ye ayit miser | W | Herb | Leaf | F | HU | "To soften palm" | Leaf is crushed and rubbed it on palm | Creaming | Dermal |  | HA 016 |
| *Bersama abyssinica* Fresen. | Melianthaceae | Kazmire | H | Tree | Leaf | F | HU | Constipation | Leaf is boiled and drunk the decoction when cool with tea cup | Drinking | Oral | Fo | HA 075 |
| *Beta vulgaris* L. | Brassicaceae | Kosxa | H | Herb | Leaf | F | HU | Constipation | Leaf is cooked and then eaten with *Injera* | Eating | Oral | Fo | HA 040 |
| *Brassica carinata* A.Braun | Brassicaceae | Wofzerer | W | Herb | Leaf | F | HU | Impetigo "(*Firat*, (*Bekebk*)" | Leaf is crushed, squeezed and creamed on the infected part | Creaming | Dermal |  | HA 067 |
| *Buddleja polystachya* Fresen. | Loganiceae | Anfar | W | Shrub | Leaf | D/F | HU | Wound | Leaf is ground, powdered and applied on wound with finger line | Creaming | Dermal |  | HA 018 |
| *Calpurnia aurea* (Aiton) Benth. | Fabaceae | Digixa | W | Shrub | Seed | D | Hu | Rabies | Seed is ground and mixed with water then drunk with tea cup | Drinking | Oral |  | HA 080 |
|  |  |  |  |  | Leaf | F | Li | "*Kincham*" | The cattle body is washed with leaf | Creaming | Dermal |  |  |
|  |  |  |  |  | Leaf | F | Li | Helminthiasis | Leaf is pounded, homogenized with cold water and then drunk by the animal with can | Drinking | Oral |  |  |
|  |  |  |  |  | Leaf | F | Li | Tick infestation | Juice is extracted and painted | Creaming | Dermal |  |  |
| *Capsicum annuum* L. | Solanaceae | Berbere | H | Herb | Fruit | D | Hu | Skin rush "(*Chifee*)" | Fruit is pounded, powdered, mixed with butter and creamed on the infected parts | Creaming | Dermal | Sp | HA 052 |
|  |  |  |  |  | Fruit | F | Li | "*Agrebreb*" | Fruit is crushed, mixed with honey and creamed on the infected part of the cattle | Creaming | Dermal |  |  |
| *Capsicum frutescens* Rodsch. | Solanaceae | Mitmita | H | Shrub | Root | F/D | HU | Taeniasis | Root is boiled and drank the decoction when cool with tea cup | Drinking | Oral | S | HA 041 |
| *Carica papaya* L. | Caricaceae | Papaya | H | Tree | Seed | F | HU | Gastritis | Seed is eaten | Eating | Oral | Fo | HA 083 |
| *Carthamus tinctorius* L | Asteraceae | Suf | H | Shrub | Seed | D | HU | Cough (lv) | Seed is roosted and mixed with raw seed of *H. vulgare* and then given for donkey with can | Eating | Oral | Fo | HA 005 |
| *Catha edulis* (Vahl) Endl. | Celastraceae | Chat | H | Shrub | Leaf | F | HU | Depression | Leaf is eaten | Eating | Oral |  | HA 074 |
| *Celosia trigyna* L. | Amaranthaceae | Lemlemcho | H | Herb | Seed | D | HU | Tape worm | Seed is ground and drunk with water with coffee cup | Drinking | Oral |  | HA 056 |
| *Chenopodium murale* L. | Chenopodiaceae | Amedmado | H | Herb | Leaf | F/D | HU | Wound | Leaf is ground, squeezed and creamed the infected parts with finger line | Creaming | Dermal |  | HA 030 |
| *Cicer arietinum* L. | Fabaceae | Shinbira | H | Herb | Seed | D | HU | Ascariasis | Seed of is eaten | Eating | Oral | Fo | HA 068 |
| *Citrus aurantiifolia* (Christm.) Swingle | Rutaceae | Lomi | H | Shrub | Fruit | F | HU | Athletes foot | Fruit is squeezed and creamed on affected part for continuous days | Creaming | Dermal | Fo | HA 017 |
|  |  |  |  |  | Fruit | F | HU | Skin disease "(*Kech*)" | Fruit is creamed on affected part for continuous days | Creaming | Dermal |  |  |
|  |  |  |  |  | Fruit | F | HU | Typhoid "(*Mognbagegn*)" | Fruit is squeezed and then drank it with coffee cup | Drinking | Oral |  |  |
| *Citrus* x *aurantium* L. | Rutaceae | Lomishet | H | Shrub | Flower | F | HU | Hypertension | Juice is drunk once a day with spoon | Drinking | Oral | Fo | HA 062 |
|  |  |  |  |  | Fruit | F | HU | Cough | Juice is drunk with spoon | Drinking | Oral |  |  |
|  |  |  |  |  | Fruit | F | HU | Common cold | Juice is boiled and the decoction is drunk when cooled with coffee cup | Drinking | Oral |  |  |
|  |  |  |  |  | Fruit | F | HU | Ascariasis | Juice is drunk with coffee cup | Drinking | Oral |  |  |
| *Clutia abyssinica* Jaub. & Spach | Euphorbiaceae | Fiyel fej | H | Herb | Leaf | F | Li | Diarrhoea | Leaf is crushed, mixed with water and given for the goat | Drinking | Oral | Fr | HA 087 |
|  |  |  |  |  | Leaf | F | HU | Ear disease | Leaf is pounded, squeezed and then the liquid is dropped through ear | Droping | Auricular |  |  |
|  |  |  |  |  | Leaf | F | HU | Dandruff | Leaf is pounded, squeezed and creamed on the affected part until recovery | Creaming | Dermal |  |  |
| *Coffea arabica* L. | Rubiaceae | Buna | H | Shrub | Seed | D | HU | Diarrhoea | Seed is roasted, crushed, powdered, boiled and the filtrate one cup of tea, mixed with few drop of oil then drunk | Drinking | Oral | Fo | HA 042 |
|  |  |  |  |  | Seed | D | HU | Wound (Due to fire burn) | Seed is roasted, crushed, powdered and applied on wound with finger line | Put on | Dermal |  |  |
| *Cordia africana* Lam. | Boraginaceae | Wanza | W | Tree | Leaf | D | HU | Fire burn | Leaf is roasted, powdered and mixed with butter and creamed affected part until recovery | Creaming | Dermal | Fu, L, Co, Ch, Fe,, Fo | HA 031 |
|  |  |  |  |  | Leaf | F | HU | Expel ear mites | Leaf is rubbed then squeezed is dropped through ear tube then covered with cotton | Dropping | Auricular |  |  |
| *Crinum abyssinicum* Hochst. ex A.Rich. | Amaryllidaceae | Yejib shenkurte | H | Herb | Root | F | HU | Ear disease | Root is pounded, squeezed and then the liquid is  dropped through ear | Droping | Auricular | Fr | HA 079 |
|  |  |  |  |  | Root | F | Li | Anthrax | Root is pounded, mixed with cold water and then given to the animal to drink it | Drinking | Oral |  |  |
|  |  |  |  |  | Shoot | D | HU | Evil eye | Shoot is put on the fire and then the smoke is inhaled | Inhale | Nasal |  |  |
|  |  |  |  |  | Shoot | D | HU | "*Telkeskash* (*Leagant*)" | Shoot is put on the fire and then smoke is inhaled | Inhale | Nasal |  |  |
|  |  |  |  |  |  |  |  |  |  |  |  |  |  |
| *Croton macrostachyus* Hochst. ex Delile | Euphorbiaceae | Bisana | W | Tree | Leaf | D/ F | HU | Liver problem | Seven pieces of immature leaves are pounded, powdered and mixed with milk and *A. sativum* and then drunk with coffee cup | Drinking | Oral | L, Co,Fe,Ch | HA 009 |
|  |  |  |  |  | Sap | F | Li | Wound | Sap is extracted and applied on the infected part | Creaming | Dermal |  |  |
|  |  |  |  |  | Bark | D | HU | Ascariasis | The 1/3 of a bark is crushed, powdered, mixed with *C. arietinum* powder, water and baked and then eaten before any food | Drinking | Oral |  |  |
|  |  |  |  |  | Sap | F | HU | Fungal skin infection "(*Cheret*)" | Sap is extracted and applied on the infected part | Creaming | Dermal |  |  |
| *Cucumis ficifolius* A.Rich. | Cucurbitaceae | Yemidir enbuay | W | Climber | Root | D | HU | Stomachache | Root is chewed and swallowed during the feeling of ache | Drinking | Oral |  | HA 090 |
|  |  |  |  |  | Leaf & root | F | Li | Wound | Leaf and root are ground and then pasted on the infected part | Creaming | Dermal |  |  |
|  |  |  |  |  | Root | D | HU | Fever, headache & vomiting "(*Dengetegna*)" | Root is pounded, powdered and mixed with water and then drunk with coffee cup | Drinking | Oral |  |  |
| *Cucurbita pepo* L*.* | Cucurbitaceae | Duba | H | Climber | Fruit | F | Both | Expel placenta | Fruit is chopped and the flesh part is boiled and given to cattle, goat and sheep | Drinking | Oral | Fo | HA 032 |
|  |  |  |  |  | Fruit | F | Both | Heart and gastritis | Fruit is chopped and the flesh part is boiled and then eaten in the morning without taking other food | Drinking | Oral |  |  |
|  |  |  |  |  | Seed | D | HU | Taeniasis | Seed is dried, roasted and then eaten | Drinking | Oral |  |  |
| *Cyperus bulbosus* Vahl | Cyperaceae | Ingicha | W | Herb | Root | F | HU | Jaundice | Root is crushed, mixed with cold water and then drunk | Drinking | Oral |  | HA 102 |
| *Datura stramonium* L. | Solanaceae | Atefaris | W | Shrub | Leaf | F | HU | Eczema | Leaf is crushed and applied on the infected part | Creaming | Dermal |  | HA 057 |
| *Discopodium penninervium* Hochst. | Solanaceae | Ameraro | W | Shrub | Root | F | HU | Snake bite | Root is boiled and then the decoction is drunk when cooled | Drinking | Oral | L, Fe, | HA 014 |
|  |  |  |  |  | Leaf | F | Li | Blackleg | Leaf is pounded, mixed with cold water and then given to the animal to drink it | Drinking | Oral |  |  |
|  |  |  |  |  | Leaf | F | Li | Wound | Leaf is ground and the surface of the diseased part is covered with the powder | Creaming | Dermal |  |  |
|  |  |  |  |  | Leaf | F | HU | Hepatitis | Leaf is crushed and mixed with bat meat and eaten before taking any food without | Drinking | Oral |  |  |
| *Dodonaea angustifolia* L.f. | Sapidaceae | Kitkita | W | Tree | Leaf | D/F | HU | Wound | Leaf is ground, powdered and then applied on  wounded part | Creaming | Dermal |  | HA 103 |
|  |  |  |  |  | Leaf | D | HU | Skin rash "(*Chiffee*)" | Leaf is roasted, ground, powdered, mixed with butter and then creamed on the affected part | Creaming | Dermal |  |  |
|  |  |  |  |  | Root | F | Li | Ringworm | Root is ground and surface of the diseased part is covered with its powder | Drinking | Oral |  |  |
| *Dovyalis abyssinica* (A.Rich.) Warb. | Flacourtiaceae | Koshim | H | Shrub | Fruit | F |  | Intestinal parasites | Fruit is eaten as food for the case of intestinal parasite before breakfast every morning with coffee cup | Eating | Oral | Fo, L | HA 033 |
| *Echinops kebericho* Mesfin | Asteraceae | Keberchio | H | Shrub | Root | F/D | HU | Evil eye | Root is crushed, burnt and its smoke and seam are inhaled | Inhale | Nasal |  | HA 096 |
|  |  |  |  |  |  |  |  | Evil spirit | Root is crushed, burnt and its smoke and seam are inhaled | Inhale | Nasal |  |  |
| *Echium plantagineum* L. | Boraginaceae | Godolo kixel | H | Shrub | Whole part | F | Li | Diarrhoea | Whole part are given to cattle | Drinking | Oral | Fr | HA 019 |
|  |  |  |  |  | Whole part | D | HU | Evil eye | Whole part are put on fire and then the smoke is inhaled | Inhale | Nasal |  |  |
| *Ehretia cymosa* Thonn. | Boraginaceae | Game | W | Shrub | Leaf & root | F/D | HU | Sudden sickness (lv) | Leaf or root is crushed and mixed with water and then drunk, put on fire and fumigated | Drinking and smoking | Oral, nasal |  | HA 084 |
| *Eragrostis tef* (Zucc.) Trotter | Poaceae | Tef | H | Herb | Seed | D | HU | Dandruff '(*Forefor*)" | Seed is ground, a dough is prepared then creamed on bare head | Creaming | Dermal | Fo | HA 038 |
| *Eucalyptus globulus* Labill. | Myrtaceae | Nech bahirzaf | H | Tree | Leaf | F | HU | Fibril illness and bronchitis | Leaf is chopped and boiled with water and inhaled repeatedly the vapor | Sniff | Nasal | Fe | HA 076 |
| *Euphorbia abyssinica* J.F.Gmel. | Euphorbiaceae | Qulquale | W | Herb | Sap/ latex | D/F | HU | Ascariasis | The drop of latex is collected, mixed with “teff” powder or honey and baked and then eaten before any food | Eating | Oral | Fo | HA 070 |
|  |  |  |  |  | Bark | D | Li | Caught | Bark is put on fire and then the smoke is fumigated | Inhale | Nasal |  |  |
|  |  |  |  |  | Latex | F | HU | Dandruff | Latex is creamed on the infected part | Creaming | Dermal |  |  |
| *Euphorbia platyphyllos* L. | Euphorbiaceae | Antirfa | H | Herb | Latex | F | HU | Hemorrhoid | Milky latex is pasted on the injured parts with finger line | Creaming | Anal |  | HA 001 |
| *Foeniculum vulgare* Mill. | Apiaceae | Ensilale | H | Herb | Leaf & steam | F | Both | Diuretic | Leaf and stem are cooked and then drunk | Drinking | Oral | Fo | HA 058 |
| *Glycine max* (L.) Merr. | Fabaceae | Akuri ater | H | Shrub | Leaf | F | HU | Vomiting "(*Ankar*)' | Leaf is crushed, mixed with water and drunk with tea cup | Drinking | Oral | Fo | HA 037 |
| *Gossypium barbadense* L. | Malvaceae | Tit | H | Shrub | Fruit | D | HU | Tonsillitis | Fruit is ground and the liquid is drunk with coffee cup | Drinking | Oral |  | HA 063 |
| *Guizotia abyssinica* (L.f.) Cass. | Asteraceae | Nug | H | Herb | Seed | D/F | HU | Common cold | Seed is boiled and the decoction is drunk when cooled with teacup | Drinking | Oral | Fo | HA 020 |
| *Hagenia abyssinica* (Bruce ex Steud.) J.F.Gmel. | Rosaceae | Kosso | W | Tree | Seed | D | HU | Tape worm | Seed is crushed, powdered mixed with milk and boiled and drunk with tea cup | Drinking | Oral | Co, Si, Fu, L, Ch | HA 091 |
| *Hordeum vulgare* L. | Poaceae | Gebs | H | Herb | Seed | F | HU | Dandruff | Seed is crushed and then creamed on the affected part | Creaming | Dermal |  | HA 053 |
| *Hypericum quartinianum* A.Rich. | Guttiferae | Ameja | H | Shrub | Flower, | F | HU | Mumps | Flower juice is extracted and then painted on the infected part | Droping | Auricular |  | HA 034 |
|  |  |  |  |  | Root | F | Li | Bleeding | Root juice is extracted and then painted on the bleeding site | Creaming | Dermal |  |  |
|  |  |  |  |  | Root | F | Li | Snake bite | Root is ground, mixed with cold water and then applied on animal on the infected part | Creaming | Dermal |  |  |
|  |  |  |  |  | Leaf | F | Li | “*Fengl*" | Leaf is crushed and mixed with water, A. *sativum* and *injera* then given for hen | Eating | Oral |  |  |
|  |  |  |  |  | Leaf | D | HU | Rabies | The crushed leaf is mixed with the ground bark of A. *abyssinica* and water then drunk with coffee cup | Drinking | Oral |  |  |
| *Impatiens tinctoria* A.Rich. | Balsaminaceae | Enshoshela (Geshlet) | H | Shrub | Tuber | F | HU | Arthritis"(*Reh*)" | Tuber is crushed, boiled, squeezed then drunk with coffee cup | Drinking | Oral | So | HA 023 |
| *Inula confertiflora* A.Rich. | Asteraceae | Woynagift | H | Shrub | Leaf | F | HU | Common cold | Leaf is boiled with *A. sativum* and then the decoction is drunk when cooled with coffee cup | Drinking | Oral |  | HA 043 |
|  |  |  |  |  | Leaf | F | HU | Cough | Leaf is boiled and then decoction is drunk when cooled | Drinking | Oral |  |  |
|  |  |  |  |  | Root | F | Li | Ringworm | Juice/oil/latex is extracted and painted on the infected body with finger line | Creaming | Dermal |  |  |
|  |  |  |  |  | Leaf | F | Li | Lumpy skin disease | Leaf is pounded, mixed with cold water and given to animal to drink it | Drinking | Oral |  |  |
|  |  |  |  |  | Leaf | F | HU | Post-partum hemorrhage | Leaf is crushed, boiled with water and then the decoction is given for delivered mother after or before it happened | Drinking | Oral |  |  |
| *Juniperus procera* Hochst. ex Endl. | Cuppressaceae | Tsed | H | Tree | Seed | F | HU | Ear disease | Seed of *J. procera* with *Sesamum oriontale* is crushed mixed with little water then the solution is filtrated the and then 3-7 drops are added through ear | Dropping | Auricular | Or | HA 094 |
| *Kalanchoe marmorata* Baker | Crassulaceae | Shikrkrit | H | Herb | Leaf | F | HU | Common cold | Leaf is crushed, squeezed and then taken with coffee or tea until recovery with tea cup | Drinking | Oral |  | HA 027 |
|  |  |  |  |  | Leaf | F | HU | Cough | Leaf is crushed, squeezed and then taken with coffee or tea until recovery | Drinking | Oral |  |  |
|  |  |  |  |  | Root | F | HU | Tonsillitis | Root is chewed and the juice is swallowed | Drinking | Oral |  |  |
| *Kalanchoe petitiana* A.Rich. | Crassulaceae | Endahula | H | Herb | Leaf | F | HU | Body swelling | Leaf is squeezed with water and creamed on swell part | Creaming | Dermal |  | HA 098 |
|  |  |  |  |  | Root | F | HU | Tonsillitis | Root is crushed, squeezed in the water and one cup is taken orally | Drinking | Oral |  |  |
|  |  |  |  |  | Leaf | F | HU | Wound | Leaf is crushed and put on the infected part | Creaming | Dermal |  |  |
| *Lagenaria siceraria* (Molina) Standl. | Cucurbitaceae | Qel | H | Climber | Fruit | D | HU | Abscess | Fruit is first heated and then put on swelling part of the body | Put on |  |  | HA 065 |
| *Laggera tomentosa* (Sch.Bip. ex A.Rich.) Oliv. & Hiern | Asteraceae | Kesekeso | H | Shrub | Root | F | HU | Hemorrhoids | Root is ground and infected part is covered with it | Creaming | Anal | Tb | HA 100 |
| *Lens culinaris* Medik. | Fabaceae | Meser | H | Herb | Seed | D | HU | Spider poison | Seed is swallowed and then expel in to the infected part | Expel | Dermal | Fo | HA 021 |
| *Leonotis ocymifolia* (Burm.f.) Iwarsson | Lamiaceae | Ras kimir | H | Shrub | Leaf | F | HU | Nausea | Leaf is crushed, mixed with cold water and it is drunk with coffee cup | Drinking | Oral |  | HA 101 |
|  |  |  |  |  | Root | F | Li | Anthrax | Root is ground, mixed with cold water and given for animal | Drinking | Oral |  |  |
|  |  |  |  |  | Leaf | F | Li | Blackleg | Leaf is ground, mixed with cold water and then given for animal with can | Drinking | Oral |  |  |
|  |  |  |  |  | Leaf | D | HU | Tonsillitis | Leaf is burnt with fire and ground then taken orally | Eating | Oral |  |  |
|  |  |  |  |  | Leaf | F | HU | Sudden sickness | Leaf is crushed and mixed with water and then it is drunk | Drinking | Oral |  |  |
|  |  |  |  |  | Leaf | F | HU | Ascariasis | Leaf is crushed, squeezed and drunk with spoon | Drinking | Oral |  |  |
|  |  |  |  |  | Leaf | F | HU | Headache | Leaf is crushed, squeezed, mixed with *O. lamiifolium* and drunk with coffee or tea with coffee cup | Drinking | Oral |  |  |
| *Lepidium sativum* L. | Brassicaceae | Feto | H | Herb | Seed | F/D | HU | Malaise | Seed is boiled and the decoction is drunk when cooled with teacup | Drinking | Oral |  | HA 071 |
|  |  |  |  |  | Seed |  | HU | Gastritis | Seed is boiled and drank the decoction when cool with coffee cup | Drinking | Oral |  |  |
|  |  |  |  |  | Stem | F | HU | Hemorrhoids | Stem is first heated and then put on the infected part of the body until recovery | Put on | Dermal |  |  |
|  |  |  |  |  | Leaf &seed | F |  | Sudden sickness | Leaf and seed are boiled and the decoction is drunk when cooled with coffee cup | Drinking | Oral |  |  |
|  |  |  |  |  | Seed | D | HU | "*Telkeskash* (*Leagant*)" | Seed is put on fire and the smoke is inhaled | Sniff | Nasal |  |  |
| *Linum usitatissimum* L*.* | Linaceae | Tilba | H | Herb | Seed | D | HU | Retained placenta | Seed is mixed water and boiled and then the cooled solution is drunk | Drinking | Oral | Fo | HA 036 |
|  |  |  |  |  | Seed | D | Li | Gastric | Seed is crushed, powdered, mixed water and sugar and then drunk during feeling pain | Drinking | Oral |  |  |
| *Lycopersicon esculentum* Mill. | Solanaceae | Timatim | H | Herb | Leaf | F | HU | Leech (lv) | Leaf is chopped then given for cattle | Eating | Oral | Fo | HA 086 |
| *Maesa lanceolata* Forssk. | Myrsinaceae | Kelewa | W | Tree | Root | F/D | HU | Constipation | Root is boiled and the decoction is drunk when cooled | Drinking | Oral | L, Fu,Ch,Co,Fe | HA 024 |
|  |  |  |  |  | Fruit | F | HU | Taeniasis | Crush, homogenize with cold water and drink | Drinking | Oral |  |  |
|  |  |  |  |  | Leaf | F | Li | Tick infestation | Extract the juice/oil/latex and pour it | Eating | Oral |  |  |
|  |  |  |  |  | Fruit | F | Li | Parasitic Leech | Pound, homogenize with cold water and allow the animal to drink it or pour it on infected part | Drinking | Oral |  |  |
| *Momordica foetida* Schumach. | Cucurbitaceae | Yamora misa | W | Shrub | Root | F | HU | Gastritis | Root is boiled and the decoction is drunk when cooled with coffee cup | Drinking | Oral |  | HA 085 |
|  |  |  |  |  | Root | F | HU | Constipation | Root is boiled and the decoction is drunk when cooled with coffee cup | Drinking | Oral |  |  |
|  |  |  |  |  | Root, Leaf | F | HU | Fever | Root and leaf are boiled and the steam is inhaled | Sniff | Nasal |  |  |
| *Myrsine africana* L. | Myrsinaceae | Kechemo | W | Tree | Fruit | F | HU | Diabetes mellitus | Crush, homogenize with cold water and drink with spoon | Drinking | Oral |  | HA 029 |
| *Myrtus communis* L. | Myrtaceae | Ades | H | Shrub | Leaf | D | HU | Dandruff | Leaf is powdered, mixed with butter and creamed on affected part | Creaming | Dermal |  | HA 099 |
| *Nicotiana tabacum* L. | Solanaceae | Timbaho | H | Herb | Leaf | F | HU | Leech (lv) | Leaf is pounded, squeezed and given for cattle | Eating | Oral |  | HA 064 |
| *Nigella sativa* L. | Ranunculaceae | Tekure azemude | H | Herb | Seed | D/F | HU | Asthma | Drink the concoction with spoon | Drinking | Oral |  | HA 095 |
| *Ocimum lamiifolium* Hochst. ex Benth. | Lamiaceae | Damakessie | H | Shrub | Leaf | F | HU | Febrile illness | Fresh leaf is squeezed and added in tea or coffee and drunk with coffee cup | Drinking | Oral |  | HA 003 |
|  |  |  |  |  | Leaf | F | Li | Amoebiasis | Leaf is ground, mixed with cold water and given to animal to drink it or poured on the infected part | Drinking | Oral |  |  |
|  |  |  |  |  | Leaf | F | Li | Poor appetite | Leaf is ground, mixed with cold water and then given to the animal to drink it | Drinking | Oral |  |  |
|  |  |  |  |  | Leaf | F | HU | Headache | Leaf is crushed, squeezed and then drunk | Drinking | Oral |  |  |
| . |  | Damakessie | H | Shrub | Leaf | F | HU | Febrile illness | Fresh leaf is squeezed and added in tea or coffee and drunk with coffee cup | Drinking | Oral |  | HA 003 |
|  |  |  |  |  | Leaf | F | Li | Amoebiasis | Leaf is ground, mixed with cold water and given to animal to drink it or it is poured on the infected part | Drinking | Oral |  |  |
|  |  |  |  |  | Leaf | F | Li | Poor appetite | Leaf is ground, mixed with cold water and then given to the animal to drink it | Drinking | Oral |  |  |
| *Olea europaea* L. | Oleaceae | Weyra | H | Tree | Leaf | F | HU | Tonsillitis | Leaf is chewed and juice is swallowed until recovery | Drinking | Oral | Tb | HA 077 |
|  |  |  |  |  | Root | F | HU | Lumpy skin disease | Root is ground and the powder is creamed on the infected part | Creaming | Dermal |  |  |
|  |  |  |  |  | Stem | F | HU | Toothache | Stem is chewed with teeth until recovery | Put on | Oral |  |  |
| *Otostegia integrifolia* Benth. | Lamiaceae | Tungut | H | Shrub | Leaf | F | Hu & Li | Rabies | Leaf is ground mixed with milk and drunk | Drinking | Oral |  | HA 059 |
| *Persea americana* Mill. | Lauraceae | Avocado | H | Shrub | Fruit | F | HU | Constipation | Fruit is eaten without processing | Eating | Oral | Fo | HA 035 |
| *Phytolacca dodecandra* L’Herit. | Phytolaccaceae | Indod | W | Shrub | Root | D | HU | Gonorrhea | Root is boiled and then the decoction is drunk when cooled | Drinking | Oral |  | HA 092 |
|  |  |  |  |  | Leaf | F | Li | Lice infestation in chicken | Extract the latex and give it for hen | Drinking | Oral |  |  |
|  |  |  |  |  | Leaf | F | Li | Parasitic Leech | Leaf is ground, mixed with cold water and given for animal | Drinking | Oral |  |  |
|  |  |  |  |  | Root | F | Li | Helminthiasis | Root is ground, mixed with cold water and given for animal | Drinking | Oral |  |  |
|  |  |  |  |  | Leaf | F | HU | To prevent pregnancy | A small part fresh leaf is chewed | Drinking | Oral |  |  |
| *Pisum sativum* L. | Fabacea | Ater | H | Herb | Seed | D | HU | Cellulites | Seed is crushed and then placed on the swelling part until the abscess site brings eye like structure or burst with line | Tied | Dermal | Fo | HA 026 |
| *Plectranthus cylindraceus* Hochst. ex. Benth. | Lamiaceae | Tibtibo | H | Herb | Leaf | F | HU | Febrile illness "(*Mich*)" | Leaf is crushed and then the whole body is drunk after baking *injera* | Rubbing | Dermal |  | HA 050 |
| *Prunus persica* (L.) Batsch | Rosaceae | Kok | H | Shrub | Leaf | F | HU | Diarrhoea (lv) | Leaf is crushed and immersed in water for few minutes and then given to cattle | Eating | Oral | Fo | HA 044 |
| *Psidium guajava* L. | Myrtaceae | Zeytune | H | Tree | Fruit | F | HU | Amoebiasis | Seed is eaten | Eating | Oral | Fo | HA 093 |
| *Pterolobium stellatum* (Forssk.) Brenan | Fabaceae | Kontir | W | Shrub | Root | F | HU | Evil eye | Root is crushed, boiled the part and inhale its steam | Sniff | Nasal |  | HA 049 |
| *Rhamnus prinoides* L’Herit | Rhamnaceae | Gesho | H | Shrub | Shoot | F | HU | Tonsillitis for children | Shoot is ground, mixed with water and drunk with spoon | Drinking | Oral | Tb | HA 073 |
|  |  |  |  |  | Leaf | F | HU | Herpes "(*Almaz-Balechira*)" | Leaf is ground and creamed on the wound | Creaming | Dermal |  |  |
| *Ricinus communis* L. | Euphorbiaceae | Gulo | H | Shrub | Fruit | F | HU | Hemorrhoids | The oil is extracted from the fruit part and painted on the infected part | Painting | Anal | Fo | HA 006 |
|  |  |  |  |  | Fruit | F | Li | Scabies | The oil is extracted from the fruit part and drunk with can | Drinking | Oral |  |  |
| *Rosa abyssinica* R.Br. | Rosaceae | Kega | W | Shrub | Root | F | HU | Constipation | Root is boiled and the decoction is drunk when cooled with teacup | Drinking | Oral | Fo | HA 089 |
| *Rumex nepalensis* Spreng*.* | Polygonaceae | Tult | H | Herb | Root | F | Li | Anthrax | Root is crushed and given with water for cattle | Eating | Oral | Fr | HA 045 |
|  |  |  |  |  | Root | D/F | Both | Stomach ache | Root is chewed and its juice is swallowed | Swallowing | Oral |  |  |
|  |  |  |  |  | Root | D/F | HU | Tonsillitis | Root is crushed, mixed with water and small amount is tied on neck | Tied | Dermal |  |  |
|  |  |  |  |  | Root | D/F | HU | Sudden sickness | Root is chewed and its juice is drunk | Drinking | Oral |  |  |
|  |  |  |  |  | Root | F/D | HU | Diarrhoea | Root is boiled and the decoction is drunk when cooled | Drinking | Oral |  |  |
|  |  |  |  |  | Root | F | Both | Abdominal plotting | Root is crushed and mixed with water and then given for cattle, for human | Swallowing | Oral |  |  |
|  |  |  |  |  | Root | F | HU | Placenta expulsion | Root of *R. nepalensis* and leaf of *J. procera* are crushed and mixed with water then drunk | Drinking | Oral |  |  |
| *Rumex nervosus* Vahl | Polygonaceae | Embuacho | H | Herb | Leaf | F | HU | Wound | Leaf is pounded, squeezed and then creamed on wound | Creaming | Dermal |  | HA 082 |
|  |  |  |  |  | Root | F | HU | Tooth pain | Root is chewed | Put on | Oral |  |  |
|  |  |  |  |  | Leaf | F | HU | Stop bleeding for men | Leaf is crushed and hold the bleeding site with it after circumcision | Put on | Dermal |  |  |
| *Ruta chalepensis* L. | Rutaceae | Tenadam | H | Shrub | Leaf | F | HU | Stomachache | Leaf is crushed, Squeezed and the juice is drunk during ache with spoon | Drinking | Oral | Sp | HA 066 |
| *Salvia nilotica* Juss. Ex Jacq. | Lamiaceae | Hullegeb | H | Herb | Leaf | F | HU | Febrile illness "(*Mich*)" | Leaf is crushed and then the whole body is rubbed | Rubbing | Dermal |  | HA 010 |
| *Satureja simensis* (Benth.) Briq. | Lamiaceae | Deresh benebse | H | Herb | Leaf | F | HU | Headache, Sudden sickness and high fever "(*Mich*)" | Leaf is crushed and mixed with water then drunk and smoked | Drinking, Sniff | Oral, nasal & dermal |  | HA 048 |
| *Schinus molle* L. | Anacerdiaceae | Kundo berbere | H | Tree | Seed | F | HU | Tonsillitis | Seed is chewed | Swallowing | Oral | Sp | HA 097 |
| *Senecio myriocephalus* Sch. Bip. ex A.Rich. | Asteraceae | Sibut | H | Shrub | Leaf | F | HU | Wound | Leaf is crushed and creamed on wound | Creaming | Dermal |  | HA 060 |
|  |  |  |  |  | Root | F | Li | Depression, high fever and nasal discharge | Root is ground, mixed with cold water and given for animal | Drinking | Oral |  |  |
| *Solanum incanum* L. | Solanaceae | Imbuay | W | Shrub | Leaf | F | HU | Epistaxis | Leaf is crushed and then added through the nose | Added | Nasal |  | HA 015 |
|  |  |  |  |  | Fruit | F | HU | Tonsillitis | Root is chewed and its juice is swallowed | Swallowing | Oral |  |  |
|  |  |  |  |  | Seed | F | Hu& LI | Leech | Seed is pounded. squeezed and added through nose | Addedthrough the nose | Nasal |  |  |
|  |  |  |  |  | Fruit | F | HU | Abdominal pain | Fruit is eaten |  | Oral |  |  |
| *Solanum nigrum* L. | Solanaceae | Awux | H | Herb | Fruit | F | HU | Eczema | Leaf is crushed and rubbed on infected part | Rubbing | Dermal |  | HA 081 |
|  |  |  |  |  | Fruit | F | HU | Itching | Leaf is crushed and rubbed on infected part | Rubbing | Dermal |  |  |
|  |  |  |  |  | Leaf | F | HU | Spider poison | Leaf is crushed with water and then creamed on the infected part | Creaming | Dermal |  |  |
| *Tamarindus indica* L. | Fabaceae | Miserich | H | Tree | Leaf | D/F | HU | Wound | Leaf is pounded, powdered and applied on the wound | Creaming | Dermal |  | HA 047 |
| *Thymus schimperi* Ronniger | Lamiaceae | Tosigne | H | Herb | Leaf | F/D | HU | Hypertension | Leaf is boiled and the decoction is drunk when cooled with coffee cup | Drinking | Oral | Sp | HA 072 |
|  |  |  |  |  | Leaf | F/D | HU | DM | Leaf is boiled and the decoction is drunk when cooled with coffee cup | Drinking | Oral |  |  |
| Trigonella foenum-graecum L. | Fabaceae | Abesh | H | Herb | Seed | D | HU | Gastritis | Seed is crushed, powdered, mixed with water then waited for one day and eaten | Eating | Oral | Fo | HA 007 |
|  |  |  |  |  | Seed | D | HU | Peptic ulcer disease | Seed is crushed, powdered, mixed with water then waited for one day and eaten | Eating | Oral |  |  |
| *Urtica simensis* Hochst. ex A.Rich. | Urticaceae | Sama | H | Herb | Leaf | F | HU | Hemorrhoids | Leaf is pounded, squeezed and then creamed on the affected part | Creaming | Dermal | Fo | HA 078 |
|  |  |  |  |  | Leaf | F | HU | Gastritis | Fresh leaf IS roasted like “*Wot*” and eaten by injera | Eating | Oral |  |  |
|  |  |  |  |  | Leaf | F | Li | Weight loss "*Kesat*" | Fresh leaf given for the cattle | Eating | Oral |  |  |
|  |  |  |  |  | Leaf | F | HU | Dyspepsia "(*Kar*)" | Leaf is crushed, squeezed in water and drunk with coffee cup until recovery | Drinking | Oral |  |  |
|  |  |  |  |  | Root | D | HU | Evil eye | Root is put on fire and then the smoke is inhaled | Inhale | Nasal |  |  |
|  |  |  |  |  | Leaf | F | HU | Constipation | Fresh leaf is roasted like “*wot*” and eaten by injera | Eating | Oral |  |  |
| *Verbascum sinaiticum* Benth. | Scrophulariaceae | Ye ahya joro | H | Herb | Root | F | HU | Abdominal pain | Root is ground and mixed with water then drunk | Drinking | Oral |  | HA 051 |
|  |  |  |  |  | Leaf | D | HU | Sudden sickness | Leaf is put on fire and the smoke is inhaled | Inhale | Nasal |  |  |
|  |  |  |  |  | Leaf | F | Li | Anthrax | Leaf is mixed with leaf of *R. nepalensis* and then given for cattle | Eating | Oral |  |  |
| *Vernonia amygdalina* Del. | Asteraceae | Girawa | H | Shrub | Leaf | F | HU | Athletes foot | Leaf is crushed, squeezed and creamed on the affected part for continuous days | Creaming | Dermal | Tb | HA 028 |
|  |  |  |  |  | Leaf | F | Li | Anthrax | LeaF is given for cattle | Eating | Oral |  |  |
|  |  |  |  |  | Root | F | HU | Abdominal pain | Root is chewed and its juice is swallowed | Swallowing | Oral |  |  |
| *Vernonia auriculifera* Hiern | Asteraceae | Gusho | H | Herb | Root | F | HU | Sudden sickness | Root is chewed and the juice is swallowed | Swallowing | Oral |  | HA 054 |
| *Vicia faba* L. | Fabaceae | Bakela | H | Herb | Seed | D | HU | Cellulites | Seed is tied on the swelling part until the abscess site brings eye like structure or burst | Put on | Dermal | Fo | HA 046 |
| *Withania somnifera* (L.) Dunal | Solanaceae | Gizawa | H | Shrub | Root | D/F | Li | Blackleg | Root is ground and mixed with cold water and given to cattle | Drinking | Oral |  | HA 022 |
|  |  |  |  |  | Root | D | Hu | Evil eye | Root is put on fire and the smoke is inhaled | Inhale | Nasal |  |  |
|  |  |  |  |  | Root | D | Hu | Sudden sickness | Root is put on fire and the smoke is inhaled | Inhale | Nasal |  |  |
| *Zingiber officinale* Roscoe | Zingiberaceae | Zengibel | H | Herb | Rhizome | D/F | Li | Amoebiasis | Rhizome is ground, mixed with cold water and given for animal | Drinking | Oral | Sp | HA 013 |
|  |  |  |  |  | Rhizome | D/F | Hu | Cough | Rhizome is crushed, mixed with cold water and drunk with tea cup | Drinking | Oral |  |  |
|  |  |  |  |  | Rhizome | D/F | Hu | Diarrhoea | Rhizome is boiled and the decoction is drunk when cooled | Drinking |  |  |  |
|  |  |  |  |  | Rhizome | D/F | Hu | Common Cold | Rhizome is boiled and the decoction is drunk the when cooled | Drinking | Oral |  |  |
|  |  |  |  |  | Rhizome | D/F | Li | Poor appetite | Rhizome is ground, mixed with cold water and given for animal | Drinking | Oral |  |  |
|  |  |  |  |  | Rhizome | D/F | Li | Giardiasis | Rhizome is ground, mixed with cold water and given for animal | Drinking | Oral |  |  |
| *Ziziphus spina-christi* (L.) Desf. | Rhamnaceae | Geba | W | Shrub | Leaf | D | Hu | Dandruff | Leaf is pounded, powdered, mixed with butter then creamed on the affected part | Creaming | Dermal | Fo | HA 004 |
|  |  |  |  |  | Root | F | Hu | To stop diarrhoea | Root is chewed and juice is swallowed | Drinking | Oral |  |  |
