# Supplementary material for: Ethnobotanical study of medicinal plants in Asagirt District, Northeastern Ethiopia
Source: Trop Med Health. 2023 Jan 9;51:1. doi: 10.1186/s41182-023-00493-0 (PMC9827656; doi:10.1186/s41182-023-00493-0)
Supplement: Supplementary file 2 — Additional file 2. Methods used in the preparation of remedies. [file 41182_2023_493_MOESM2_ESM.docx]

**Additional file 2** Methods used in the preparation of remedies

| Method of preparation | Number | Percent |
| --- | --- | --- |
| Crushing | 21 | 20.3 |
| Squeezing | 13 | 12.6 |
| Decoction | 9 | 8.7 |
| Grinding | 9 | 8.7 |
| Powdering and mixing | 8 | 0.7 |
| Chewing | 4 | 0.3 |
| Extract | 4 | 0.3 |
| Grinding and mixing | 3 | 0.2 |
| Chopping | 2 | 0.19 |
| Concoction | 2 | 0.19 |
| Mixing | 2 | 0.19 |
| Powdering and mixing | 2 | 0.19 |
| Roosting | 2 | 0.19 |
| Smoking | 2 | 0.19 |
| Boiling and mixing | 1 | 0.09 |
| Cooking | 1 | 0.09 |
| Crushing and mixing | 1 | 0.09 |
| Crushing, chewing and squeezing | 1 | 0.09 |
| Decoction and cover surface | 1 | 0.09 |
| Decoction, chewing and crushing | 1 | 0.09 |
| Extracting and decoction | 1 | 0.09 |
| Heating | 1 | 0.09 |
| Powdering | 1 | 0.09 |
| Smoking, crushing and squeezing | 1 | 0.09 |
| Grinding and decoction | 1 | 0.09 |
| Homogenizing and decoction | 1 | 0.09 |
| Mixing and squeezing | 1 | 0.09 |
| Mixing, squeezing and smoking | 1 | 0.09 |
| Smoking and chewing | 1 | 0.09 |
| Smoking and mixing | 1 | 0.09 |
| Smoking, roosting and squeezing | 1 | 0.09 |
| Squeezing and grinding | 1 | 0.09 |
| Total | 103 | 100 |
