# Supplementary material for: Ethnobotanical study of medicinal plants in Asagirt District, Northeastern Ethiopia
Source: Trop Med Health. 2023 Jan 9;51:1. doi: 10.1186/s41182-023-00493-0 (PMC9827656; doi:10.1186/s41182-023-00493-0)
Supplement: Supplementary file 3 — Additional file 3. Direct matrix ranking of six medicinal plant species by four informants based on seven use criteria. [file 41182_2023_493_MOESM3_ESM.docx]

**Additional file 3** Direct matrix ranking of six medicinal plant species by four informants based on seven use criteria (5 = best; 4 = Very good; 3 = good; 2 = less used; 1 = least used and 0 = no value).

| Use category | Medicinal plants | | | | | | | | | | | | | | | | | | | | | | | | |
| --- | --- | --- | --- | --- | --- | --- | --- | --- | --- | --- | --- | --- | --- | --- | --- | --- | --- | --- | --- | --- | --- | --- | --- | --- | --- |
|  | *Acacia abyssinica* Benth. | | | | *Croton macrostachyus* Hochst. ex Delile | | | | *Cordia africana* Lam. | | | | *Discopodium penninervium* Hochst. | | | | *Maesa lanceolata* Forssk. | | | | *Afrocarpus falcatus* (Thunb.) C.N.Page | | | |  |
|  | Informants (I1-4) | | | | I | | | | I | | | | I | | | | I | | | | I | | | |  |
|  | 1 | 2 | 3 | 4 | 1 | 2 | 3 | 4 | 1 | 2 | 3 | 4 | 1 | 2 | 3 | 4 | 1 | 2 | 3 | 4 | 1 | 2 | 3 | 4 |  |
| Charcoal | 5 | 4 | 3 | 2 | 4 | 3 | 5 | 4 | 4 | 4 | 3 | 5 | 0 | 1 | 2 | 3 | 3 | 2 | 2 | 3 | 5 | 4 | 3 | 4 |  |
| Furniture | 4 | 3 | 3 | 3 | 3 | 4 | 3 | 3 | 5 | 5 | 4 | 5 | 1 | 2 | 3 | 4 | 2 | 3 | 4 | 3 | 3 | 4 | 5 | 2 |  |
| Medicine | 2 | 1 | 2 | 2 | 4 | 3 | 5 | 2 | 3 | 4 | 5 | 4 | 3 | 5 | 5 | 4 | 5 | 5 | 4 | 5 | 3 | 2 | 3 | 2 |  |
| Firewood | 2 | 3 | 2 | 3 | 4 | 4 | 3 | 3 | 3 | 3 | 5 | 4 | 2 | 3 | 2 | 1 | 3 | 4 | 3 | 5 | 2 | 3 | 4 | 2 |  |
| Fencing | 1 | 2 | 2 | 2 | 3 | 2 | 3 | 4 | 3 | 4 | 5 | 4 | 5 | 4 | 3 | 2 | 3 | 2 | 3 | 1 | 2 | 3 | 4 | 3 |  |
| Construction | 4 | 4 | 3 | 5 | 4 | 3 | 3 | 4 | 5 | 5 | 4 | 5 | 0 | 1 | 0 | 2 | 4 | 3 | 2 | 2 | 4 | 4 | 5 | 4 |  |
| Fodder | 4 | 3 | 4 | 2 | 2 | 3 | 1 | 2 | 5 | 5 | 5 | 4 | 3 | 2 | 1 | 1 | 2 | 3 | 2 | 2 | 1 | 2 | 2 | 121 |  |
| Ind. total | 22 | 20 | 19 | 19 | 24 | 22 | 23 | 22 | 28 | 30 | 31 | 31 | 14 | 18 | 16 | 17 | 22 | 22 | 20 | 21 | 20 | 22 | 26 | 18 |  |
| Grand total | 80 | | | | 91 | | | | 120 | | | | 65 | | | | 85 | | | | 86 | | | |  |
| Rank | 5^th^ | | | | 2^nd^ | | | | 1^st^ | | | | 6^th^ | | | | 4^th^ | | | | 3^rd^ | | | |  |
